# Cariogenic potential of sweet flavors in electronic-cigarette liquids

**DOI:** 10.1371/journal.pone.0203717

**Published:** 2018-09-07

**Authors:** Shin Ae Kim, Samuel Smith, Carlos Beauchamp, Yang Song, Martin Chiang, Anthony Giuseppetti, Stanislav Frukhtbeyn, Ian Shaffer, Joshua Wilhide, Denis Routkevitch, John M. Ondov, Jeffrey J. Kim

**Affiliations:** 1 Volpe Research Center, American Dental Association Foundation, Gaithersburg, Maryland, United States of America; 2 Department of Chemical and Biological Engineering, University of Colorado Boulder, Boulder, Colorado, United States of America; 3 National Institute of Standards and Technology, U.S. Department of Commerce, Gaithersburg, Maryland, United States of America; 4 Molecular Characterization and Analysis Complex, University of Maryland, Baltimore County, Baltimore, Maryland, United States of America; 5 Department of Biomedical Engineering, Johns Hopkins University, Baltimore, Maryland, United States of America; 6 Department of Chemistry and Biochemistry, University of Maryland, College Park, Maryland, United States of America; University of California San Diego School of Medicine, UNITED STATES

## Abstract

**Background:**

Most electronic-cigarette liquids contain propylene glycol, glycerin, nicotine and a wide variety of flavors of which many are sweet. Sweet flavors are classified as saccharides, esters, acids or aldehydes. This study investigates changes in cariogenic potential when tooth surfaces are exposed to e-cigarette aerosols generated from well-characterized reference e-liquids with sweet flavors.

**Methods:**

Reference e-liquids were prepared by combining 20/80 propylene glycol/glycerin (by volume fraction), 10 mg/mL nicotine, and flavors. Aerosols were generated by a Universal Electronic-Cigarette Testing Device (49.2 W, 0.2 Ω). *Streptococcus mutans* (UA159) were exposed to aerosols on tooth enamel and the biological and physiochemical parameters were measured.

**Results:**

E-cigarette aerosols produced four-fold increase in microbial adhesion to enamel. Exposure to flavored aerosols led to two-fold increase in biofilm formation and up to a 27% decrease in enamel hardness compared to unflavored controls. Esters (ethyl butyrate, hexyl acetate, and triacetin) in e-liquids were associated with consistent bacteria-initiated enamel demineralization, whereas sugar alcohol (ethyl maltol) inhibited *S*. *mutans* growth and adhesion. The viscosity of the e-liquid allowed *S*. *mutans* to adhere to pits and fissures. Aerosols contained five metals (mean ± standard deviation): calcium (0.409 ± 0.002) mg/L, copper (0.011 ± 0.001) mg/L, iron (0.0051 ± 0.0003) mg/L, magnesium (0.017 ± 0.002) mg/L, and silicon (0.166 ± 0.005) mg/L.

**Conclusions:**

This study systematically evaluated e-cigarette aerosols and found that the aerosols have similar physio-chemical properties as high-sucrose, gelatinous candies and acidic drinks. Our data suggest that the combination of the viscosity of e-liquids and some classes of chemicals in sweet flavors may increase the risk of cariogenic potential. Clinical investigation is warranted to confirm the data shown here.

## Introduction

Electronic-cigarette (e-cigarette) use has steadily increased in prevalence over the past decade especially among millennials. E-cigarettes are now the most used tobacco product among U.S. middle- and high-school students, surpassing combustible cigarettes [1, 2]. The success of the e-cigarette industry, in part, can be attributed to its target market strategy to younger age group, the public’s perception that e-cigarettes are a safer alternative to traditional tobacco products, and readily available Do-It-Yourself (DIY) instructions and starter kits on social media platforms [3, 4]. E-liquids are available in a wide variety of candy-, beverage-, and fruit- like flavors, as well as traditional flavors such as tobacco and menthol [5]. E-liquids can be ordered without nicotine (AKA “pleasure without consequences”) which can be enticing to youth and young adults [6, 7]. E-cigarette use has been implicated in encouraging smoking initiation among tobacco-naïve individuals [8–10]. With passage of the 2009 Family Smoking Prevention and Tobacco Control Act, all flavors—except menthol—from conventional cigarettes have been banned in the U.S. [11]. Similarly, the European Union (E.U.) and all member states adopted E.U. Tobacco Products Directive (2014/40/EU) to prohibit characterizing flavors at the product level [12]. However, other flavored tobacco products—smokeless tobacco, little cigars and cigarillos, large cigars, hookah, dissolvables, and e-liquids—remain on the U.S., E.U., and many other global markets and continue to be readily available and prevalent [13–15].

Estimates indicate that there are over 10000 e-liquid formulations (in 2018) available from online and in-store vape shops [16]. Many research laboratories and online consumer forums have reported that the quality of currently available e-liquids varies significantly [17–23]. Inaccurate labels on products (e.g., incorrect nicotine concentration) or unintended contaminants are commonly found in commercially available e-liquids [19, 20, 23–25]. Recently, U.S. and E.U. regulations (2009 Tobacco Control Act and 2014 Tobacco Products Directive, respectively) have emphasized the need to raise e-liquid quality and manufacturing standards. The U.S. Food and Drug Administration (FDA)’s Center for Tobacco Products (CTP) issued an Advance Notice of Proposed Rulemaking (ANPRM) to obtain information related to the role that flavors play in tobacco products (Docket Number: FDA-2017-N-6565). However, internationally recognized standards (e.g., The International Organization for Standardization) on manufacturing and safety testing methods are still in an early developmental stage [26].

Although there are a wide variety of e-liquids, the basic core components of e-liquids are well-known: base, nicotine and flavors. The base is made from propylene glycol, glycerin or a mixture of the two in various ratios, diluted in purified water. The concentration of nicotine varies from 0 mg/mL to 18 mg/mL and the users typically choose their own nicotine strength. Flavors can be categorized by tastes/fragrances (e.g., bakery, beverages, fruits, menthol, and tobacco) or by their chemical compositions (e.g., saccharides, esters, acids, and aldehydes). Sucrose or sucralose is added for the sweet taste in e-liquids and sugar alcohol (e.g., ethyl maltol) is used for the sweet fragrance [27–29]. In a previous study, it was shown that the viscous base is a major cause of unintended compositional error during manufacturing and bottling processes [26]. E-liquids, especially those made from glycerin (1.412 Pa•s) have high viscosity properties. Aerosols generated from these e-liquids are likely to adhere to exposed surfaces. These surfaces include soft and hard tissues in oral cavity, nasal cavity, pharynx, epiglottis, larynx, trachea, lung (directly) and skin, hair, clothing, and indoor living spaces (indirectly). The interaction between the viscous aerosol and oral cavity is of particular interest for several reasons: (i) dental professionals have long been aware of the danger of tobacco products and nicotine on oral health, (ii) the oral cavity which includes lips, gingiva, teeth, palate and tongue is the first organ to directly interact with the e-cigarette aerosol, (iii) changes in tissue surface characteristics from eating glutinous food (e.g., caramels, licorices, or sour candies) and high sucrose intake (e.g., sodas) can lead to negative health consequences in oral cavity. Many e-liquids share similar physical and chemical properties to sugary and gelatinous foods that have been proven to be major risks for dental caries [30, 31], and recently (iv) a population-based cross-sectional study revealed that daily use of e-cigarettes is independently associated with poor oral health [32].

Although the etiological role and infectious transmission of *Streptococcus mutans* in the development of dental caries have been discovered more than 50 years ago [31], dental caries continues to be the most prevalent infectious disease in humans, affecting 97% of the world population during their lifetimes [33]. The persistence of the disease stems from the fact that dental caries cannot be attributed to a single cause. Dental caries progresses by pathogenic oral bacteria, such as *S*. *mutans*, metabolizing fermentable carbohydrate (e.g., glucose, fructose, sucrose, and maltose) to produce lactic acid [34]. At low or moderate concentration of the acid, saliva and components in saliva buffer and neutralize the low pH in oral environment [35]. However, excessive intake of sucrose disturbs the dynamic balance between pathological and protective oral factors and leads to an acidic environment where it is beyond the normal saliva buffering capacity [34]. The prolonged low pH condition promotes survival of aciduric and acidogenic bacteria such as *S*. *mutans* which have developed the ability to thrive in an acidic environment [36–38]. *S*. *mutans* also produce glucosyltransferases (GTFs) to catalyze synthesis of Intracellular Polysaccharides (IPS) and Extracellular Polymeric Substances (EPS) from sucrose [39]. These EPS significantly contribute to the formation and structural stability of oral biofilm [40]. The biofilm (AKA dental plaque) is shown to enhance attachment and protection of oral bacteria, and aid in retaining physiological nutrients including essential metal ions [41–44]. The accumulation of negative consequences and the formation of cariogenic biofilm eventually lead to break down of hard tissues (enamel and dentin) of teeth [34, 45].This study was designed to systematically evaluate whether aerosols generated from highly-characterized reference e-liquids with various sweet flavors can produce bacteria-initiated demineralization on healthy human enamel surfaces. The Universal Electronic-Cigarette Testing Machine (UECTM) was optimized to simulate human physiological parameters. A novel visualization method to quantify e-cigarette aerosol droplets and a sample preparation protocol to increase reproducibility in enamel surface adhesion measurement were developed.

## Materials and methods

### Universal electronic-cigarette testing machine (UECTM) and study design

Aerosols were generated by using a Universal Electronic-Cigarette Testing Machine (UECTM) developed by the American Dental Association (ADA) Foundation in collaboration with the University of Maryland, Department of Chemistry [26]. For all experiments, a commercial sub-ohm tank (Aspire Cleito: 0.2 Ω Kanthal coil with cotton wick) was used. Due to low resistance heating coils, sub-ohm tanks are designed to be run at higher wattages than previous generation devices. In this study, aerosols were generated at a power setting of 3.14 V (total of 49.2 W based on P = V^2^ / R) determined by the manufacturer’s manual (capable up to 55–70 W) and online “vaping power charts”. Each atomizer was used for ≤ 750 puffs (approximately 5 d usage) and replaced thereafter. If discoloration, excessive heat or abnormal sound was observed, the atomizer was immediately discarded and replaced. The entire e-cigarette system was disassembled, thoroughly cleaned with de-ionized H_2_O (diH_2_O), and dried after each experiment. Aerosols were generated using the published physiological human e-cigarette puffing topography: 50 mL puff volume in 4 s puff duration every 18 s [46]. For this study, we defined 10 puffs as one vaping session [47] and 150 puffs as one-day use (^≈^ 3mL / day) [48]. We acknowledge that no machine testing regime can represent all human vaping behavior and there is great variability across different users and devices.

### E-liquid formulation

Flavor-free reference e-liquid was prepared following our previous work (20/80 propylene glycol/glycerin (by volume fraction) with 10 mg/mL nicotine) [26]. To increase reproducibility, gravimetric method was used (0.410 g propylene glycol, 2.000 g glycerin and 20.0 mg nicotine) [26]. The following flavors were added to the reference e-liquids separately: ethyl butyrate (11.1 mg/mL); ethyl maltol (27.2 mg/mL); hexyl acetate (2.5 mg/mL); sucralose (2.0 mg/mL); and triacetin (11.6 mg/mL) (**Table 1**). The flavored e-liquids were mixed for additional 24 h using a vertical rotator at 0.5 rad/s. Selecting 20/80 propylene glycol/glycerin ratio was based on a previous study [49] and understanding that newer, high-wattage sub-ohm tanks are designed to be compatible with high glycerin e-liquids [50].

**Table 1:** Flavored reference e-liquids.

Name	Category	Taste/smell	Formula	Reported concentration (mg/mL)	Concentration used in this study (mg/mL)
**Ethyl butyrate**	Ester	Pineapple	C_6_H_12_O_2_	11.1	11.1
**Ethyl maltol**	Sugar alcohol	Cotton candy	C_7_H_8_O_3_	27.1	27.1
**Hexyl acetate**	Ester	Apple	C_8_H_16_O_2_	2.5	2.5
**Sucralose**	Sugar substitute	Sweetener	C_12_H_19_Cl_3_O_8_	1–5	2.0
**Triacetin**	Triester of glycerol and acetic acid	Velvety / smoky	C_9_H_14_O_6_	N/K	11.6

Flavors are described using their names, chemical and physical properties, and reported [28, 29] and actual concentration used in this study. The reference e-liquids are prepared using a published research on standard development [26]. Uncertainty value of measurement is ± 0.1 mg.

### Gas chromatography–mass spectrometry (GC-MS) analysis

Chemical by-product identification was performed using PerkinElmer Clarus 680 Gas Chromatography–Mass Spectrometry (GC-MS) Detection (PerkinElmer, Waltham, MA) fitted with a Velocity DB 5 column (PerkinElmer N9306325). Testing parameters of the Gas Chromatography (GC) method were as follows: Sampling method = manual headspace, Inlet temperature = 210°C, Carrier gas = 1.43 L/min, Split = 1:5, Temperature ramp = initial: 40°C, hold 3 min, 6°C/min to 300°C, hold for 3 min, and Total analysis time = 49.33 min. Testing parameters for the MS method were as follows: MS detector = PerkinElmer Clarus, ionization source = El, Polarity = positive, Mass range = (44 to 600) m/z, Acquisition type = centroid, Solvent delay = (0.00 to 2.00) min, and Analysis time = (2.00 to 49.30) min.

### Bacterial strain and culture conditions

*Streptococcus mutans* UA157 (ATCC) was used for all experiments. Frozen cells were plated on a 100 mm Brain Heart Infusion (BHI) agar plate. After overnight incubation (37 ^o^C and 5% CO_2_), a single colony was inoculated in 3 mL BHI liquid media. BHI liquid media was used for all planktonic growth assays. 75 μL of the media with bacteria was transferred to a 96 well flat-bottomed plate.

### Preparation of enamel disks

American Dental Association (ADA) Institutional Review Board has reviewed and approved the following study (MML-16-0052). Human teeth were collected during routine third molar extractions due to clinical indications, not for research purposes. Teeth were part of discarded surgical tissues and did not contain patient identifiers. Once extracted, teeth were pooled into a collection container and it was not possible for the investigators to identify the donors. Caries-free teeth were sectioned parallel to the long axis (average 5 mm thickness) and embedded in a 44 mm diameter x 5 mm thickness VariDur mounting acrylic resin (Buehler). The top of enamel disks was polished using grid 1000, 1200, 2400, and 4000 silicon carbide papers (Struers) under streaming water and which was followed by polishing with 3 μm and 1 μm sized polycrystalline diamond pastes (MetaDi, Buehler). The directionality of the disks was rotated 90^o^ in between grid changes. The quality of polishing was checked using a light metallurgical microscope (Nikon) at 40X magnification. A proper polishing step is a major component of reproducibility. The samples should not be under- or over-polished. The polished disks should have uniform orientation and be free of cracks, deformations, scratches, steps and slopes. The direction of enamel rods should be carefully considered when selecting which part of the disks is to be measured. The final disks had tooth surfaces exposed from the top and bottom. This is important to improve reproductivity in subsequent hardness measurement.

### Micro-indentation hardness tests

All measurements were performed using Knoop hardness number (KHN) following American Society for Testing and Materials (ASTM) testing methods E92-16 and E384-16. The mounted disks were placed under the Knoop indenter of a micro-indentation hardness tester (Buehler-Wilson Knoop/Vickers Hardness Tester, Tukon 1202) and subjected to a load of 50 g for 10 s. Hardness was determined at five sites between the surface of the tooth to the Dentino- Enamel Junction (DEJ). After e-cigarette exposure and subsequent bacterial attachment, another sets of KHN measurements were made on each disk on parallel tracks approximately 100 μm apart [51, 52]. The disks were thoroughly cleaned with diH2O prior to post-exposure measurement and blotted carefully with Kimwipes while avoiding desiccating the disks.

### Biofilm formation assay

To form biofilm, we used the standard O’Toole-Kolter protocol [53]. Overnight culture was inoculated in Biofilm Formation (BF) media (25% TSB + 5 mg/mL yeast extract + 30 mol/L sucrose) [54]. *S*. *mutans* were allowed to attach to the surface and collected at 4 h. Unattached cells and media were removed, and the plates were washed with diH_2_O twice. 5 mL of 0.1% crystal violet solution was added and stained the attached cells for 15 min. Crystal violet solution was removed and the plates were washed with diH_2_O twice. The plates were dried in a biological safety cabinet overnight. 5 mL of 30% acetic acid (by volume) was added and incubated at room temperature for 15 min. 75 μL of the solubilized crystal violet solution was transferred to a 96 well flat-bottomed plate. Absorbance was measured at 550 nm using a plate reader (SpectraMax, Molecular Devices).

### Scanning electron microscopy

Enamel disk samples were washed with PBS and stored at -80°C for 24 h. The samples were transferred and freeze dried in a pre-chilled lyophilizer (Freezemobile 25XL, VirTis) for 24 h. The dried samples were mounted on Scanning Electron Microscope (SEM) aluminum stubs and sputter coated in gold (Desk V HP, Denton Vacuum). The samples were imaged using SEM (JSM 5300, JEOL) with following parameters: Secondary Electron Imaging (SEI), 10.0 KV at 50X, 1,500X and 10,000X magnifications.

### Aerosol droplet quantification

A UECTM was programed with the specified physiological parameters as described in the Study Design section. Aerosols were exposed on non-reflective vinyl surface (3M Temflex 1700). The exposed surface was captured with a stereoptical light microscope (Leica MZ16). Aerosol droplets were quantified using ImageJ (NIH) “Analyze Particles” feature from three randomly chosen locations. The averaged aerosol droplet counts were compared among (0, 10 and 150) puff samples.

### Metal quantification

E-cigarette aerosol (150 puffs) was collected in 30 mL of 2% ultra-pure nitric acid using a gas condenser (Pyrex 1760–125). Inductively Coupled Plasma with Optical Emission Spectrometry (ICP-OES) analyses were performed as described previously [26]. The presence of 12 metals was evaluated: cadmium (Cd), calcium (Ca), chromium (Cr), cobalt (Co), copper (Cu), iron (Fe), lead (Pb), magnesium (Mg), manganese (Mn), nickel (Ni), palladium (Pd), and silicon (Si). The final results are shown in concentration (mg/L) after considering the dilution factor of the collecting liquid medium. Nitric acid was used during the metal quantification only.

### Atomic force microscopy

The adhesive force between *S*. *mutans* and enamel surface was measured by a single-cell force spectroscopy through the atomic force microscope (AFM, Model: Bruker BioScope Resolve). Bruker NP-O10 cantilever probe with spring constants (k) of 0.06 N m^-1^ was used for the functionalization of cantilever tip. The cantilever was immersed in 10 mmol/L Tris-HCl buffer solution (pH 8.5) containing 4mg/mL dopamine hydrochloride for 1 h. In the force spectroscopy, the dopamine-coated cantilever tip was used to attach a single *S*. *mutans* cell, then the tip was pressed against the control or aerosol exposed enamel surfaces. The adhesive force was measured by separating the cell from the enamel surface at a pulling rate of 1 Hz.

### Statistical analysis

Concentration, absorbance, adhesion force and count were quantified using mean±standard deviation (S.D.) from three independent measurements. Each experiment was performed in triplicate and was repeated at least three times. All statistical analyses were conducted using the MaxStat 3.6 statistical software (Jever-OT Cleverns, Germany). The significant level was indicated as p < 0.05 (*), p<0.005 (**), or p<0.0001 (***).

## Results

### E-cigarette deposits fine aerosol particles on surfaces

**Fig 1:**
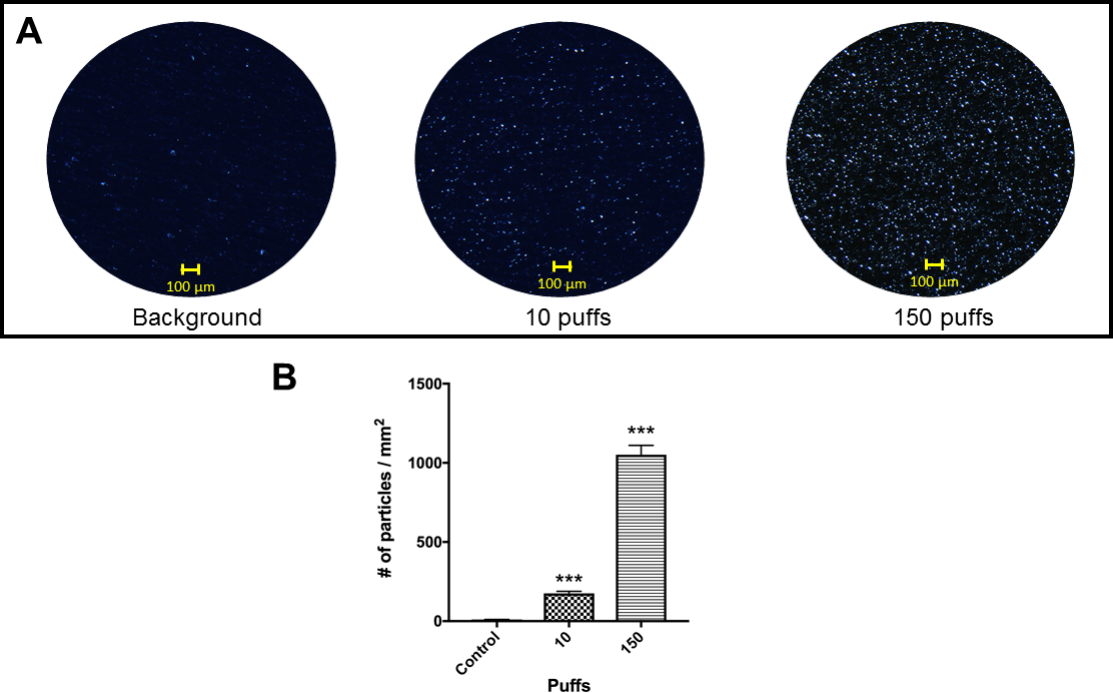
Quantification of e-cigarette aerosol droplets. **(A)** Aerosols were delivered using a simulated human vaping topology (50 mL puff volume in 4 s puff duration every 18 s). Aerosol droplets were imaged by a stereoptical light microscope on a non-reflective vinyl surface (bar = 100 μm). **(B)** ImageJ was used to quantify the aerosol droplets. The number of particles for control, after 10 puffs, and 150 puffs were (5.7 ± 5.0, 175.5 ± 12.7 and 1051.2 ± 59.4) particles per mm^2^, respectively (mean ± S.D.). Student t-tests were performed control vs. individual puffing regime (*** = p<0.0001).

To characterize e-cigarette aerosol, we used a Universal Electronic-Cigarette Testing Machine (UECTM) and reference e-liquid to generate (10 and 150) puffs, set at the predetermined physiological parameters (50 mL puff volume in 4 s puff duration every 18 s) [46]. Several surfaces were evaluated, and it was found that non-reflective vinyl surface (3M Temflex 1700) gave the least background interference when a stereoptical light microscope (Leica MZ16) was used for imaging (**Fig 1A**). Aerosols from (0, 10 and 150) puffs deposited (5.7 ± 5.0, 175.5 ± 12.7, and 1051.25 ± 59.4) aerosol particles / mm^2^ respectively (**Fig 1B**). Diameters of the visible particles ranged from 1.3 μm to 30.5 μm.

### E-cigarette generates viscous aerosol and promotes *Streptococcus mutans* attachment

**Fig 2:**
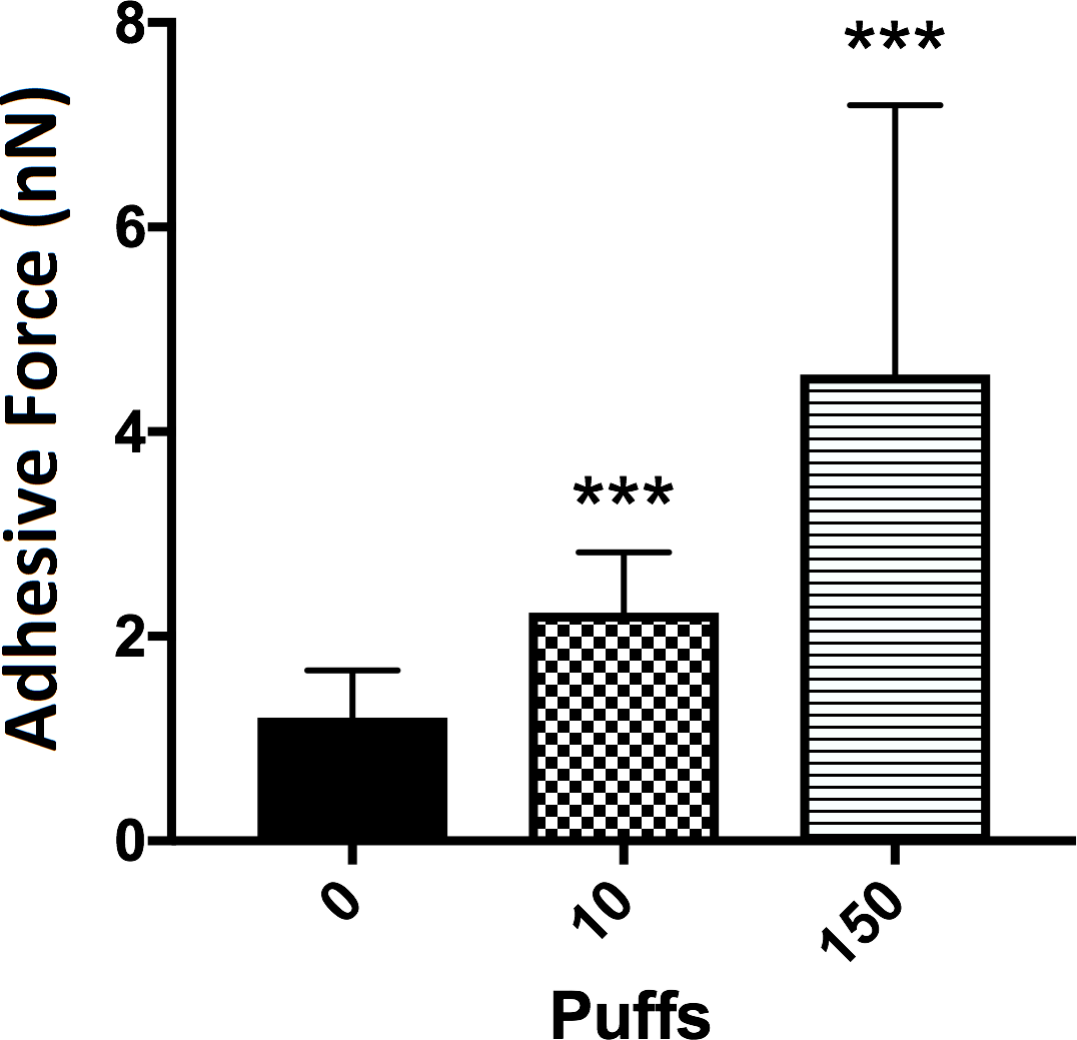
Adhesive force between *S*. *mutans* and enamel surface. The adhesive forces were calculated by averaging 30 measurements on three individual surfaces. The forces for control, after 10 puffs and 150 puffs were (1.2 ± 0.4, 2.2 ± 0.5, and 4.5 ± 2.6) nN, respectively (mean ± S.D.). Student t-tests were performed control vs. individual puffing regime (*** = p<0.0001).

To test if e-cigarette aerosol leads to a biologically-relevant surface change, human tooth enamel disks were exposed following the standard protocol as described in the Study Design. Using the single-cell force spectroscopy, the adhesive force between *S*. *mutans* and enamel surface was measured under three different conditions: control (no exposure), 10 and 150 puffs. It was found that the adhesive force between the pathogenic bacteria and enamel surface increased significantly with 10 puffs (p < 0.0001) and 150 puffs (p < 0.0001) aerosol exposure compared to the unexposed control (**Fig 2**).

### Certain flavors increase biofilm formation

**Fig 3:**
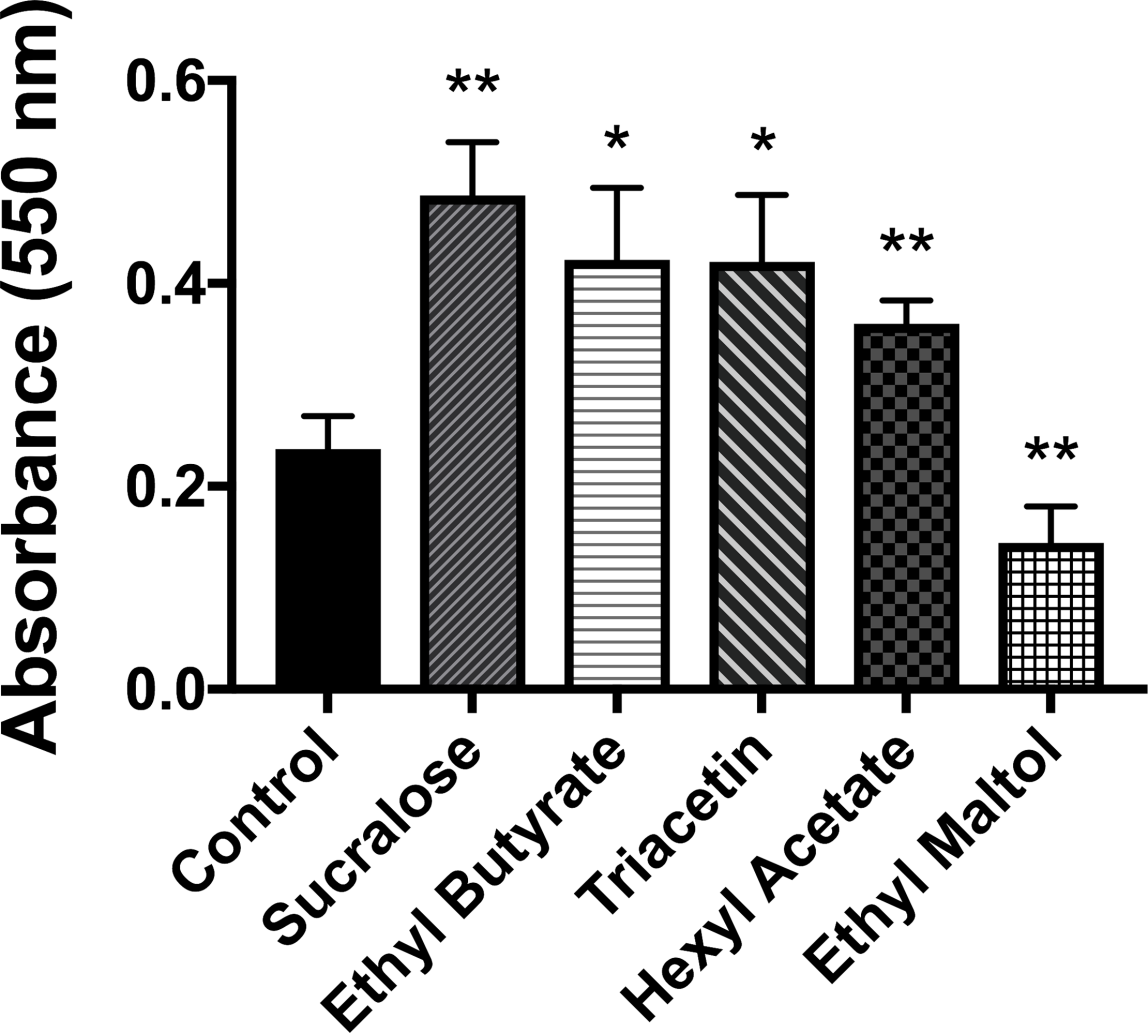
Biofilm quantification after flavored e-liquid aerosol exposures. The absorbance for control, sucralose, ethyl butyrate, triacetin, hexyl acetate and ethyl maltol were (0.23 ± 0.03, 0.48 ± 0.05, and 0.42 ± 0.07, 0.42 ± 0.06, 0.36 ± 0.02, and 0.14 ± 0.03) AU, respectively (mean ± S.D.). Student t-tests were performed control vs. individual flavored e-liquid and statistical differences were indicated as: * = p<0.05 or ** = p<0.005.

Dental plaque is a biofilm found on natural teeth. Dental plaque is implicated in dental caries which is associated with a shift in the balance of healthy oral microbiome, resulting in dysbiosis favoring disease-promoting bacteria including acid producing *S*. *mutans* [55]. The purpose of this study, therefore, was to test the effect of different e-cigarette flavor exposure to *S*. *mutans* biofilm formation. Five e-liquid flavors were pre-selected based on their high potential for cariogenicity (e.g., sweetness or low pH) and a previous study [28] which analyzed 30 commercial products: hexyl acetate (apple/plum), ethyl butyrate (pineapple), sucralose (sugar substitute), triacetin (“velvety” or “smoky” flavor) and ethyl maltol (cotton candy). Individually the five flavored e-liquids were aerosolized following the standard protocol as described in the Study Design. After forming biofilms, the amount of biofilm on each plate was quantified using the O’Toole-Kolter method. Four out of five flavors (sucralose, ethyl butyrate, triacetin, hexyl acetate) increased biofilm formation significantly compared to unflavored e-liquid control. Interestingly ethyl maltol, sugar alcohol, decreased biofilm development significantly compared to the control (**Fig 3**).

### E-cigarette aerosol occupies pits and fissures of human teeth and promotes bacterial attachment

**Fig 4:**
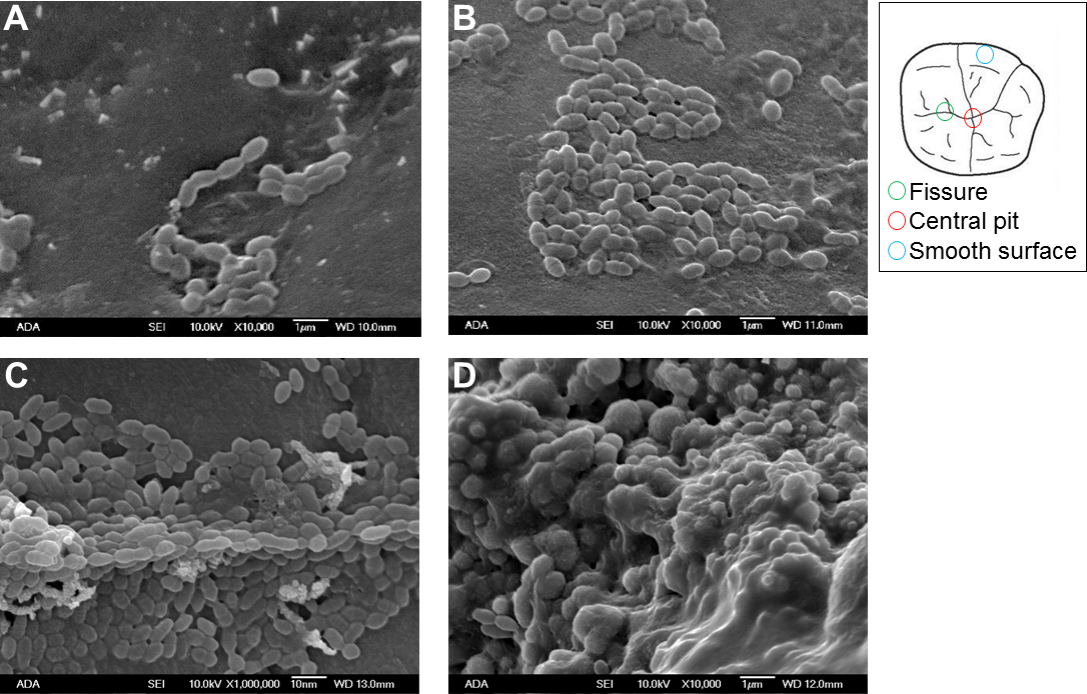
Complex interaction among *S*. *mutans*, enamel surface and e-cigarette aerosol. **(A)** Control: smooth enamel surface, unexposed. **(B)** Smooth enamel surface, exposed with 10 puff e-cigarette aerosol. **(C)** Fissure, exposed with 10 puff e-cigarette aerosol. **(D)** Central pit, exposed with 10 puff e-cigarette aerosol (SEM parameters: X10,000, 10.0 kV, and bar = 1 μm).

**Fig 5:**
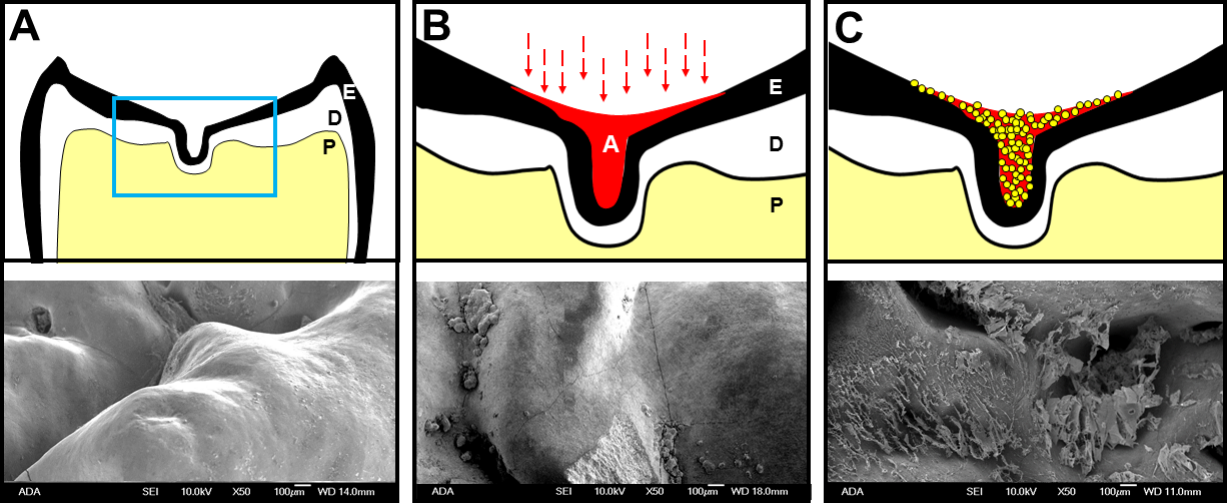
E-liquid aerosol pools into pits and fissures. **(A)** Top: a cross section of a human tooth (E = enamel, outer layer, D = dentin, middle layer, and P = pulp, cellular component with nervous and vascular tissues), Bottom: control, unexposed enamel fissure. **(B)** Top: a tooth after e-cigarette aerosol exposure (A = aerosol, E = enamel, D = dentin, and P = pulp), Bottom: aerosol exposed enamel fissure. **(C)** Top: a tooth after e-cigarette aerosol exposure and subsequent *S*. *mutans* attachment (Spheres = *S*. *mutans*), Bottom: *S*. *mutans* colonizing fissure and secreting EPS (SEM parameters: X50, 10.0 kV, and bar = 100 μm).

To characterize how e-cigarette aerosol interacts with complex biological surfaces, caries-free extracted teeth (without polishing or cutting) were exposed using the standard protocol. After a 24 h incubation with *S*. *mutans*, a Scanning Electron Microscope (SEM) was used to visualize smooth surfaces, pits, and fissures–three areas on tooth enamel surface where bacteria can attach, form a biofilm, and lead to dental caries. Generally, bacteria were found more frequently in aerosol exposed pits and fissures compared to the unexposed controls (**Fig 4**). The aerosol exposed smooth surfaces also had more *S*. *mutans* compared to the unexposed control smooth surface but to a lesser degree than pits and fissures (**Fig 4**). Once *S*. *mutans* occupied pits and fissures, the oral bacteria thrived and formed very complex biofilm by secreting EPS (**Fig 5**).

### Certain flavors demineralize enamel and decrease tooth hardness

**Fig 6:**
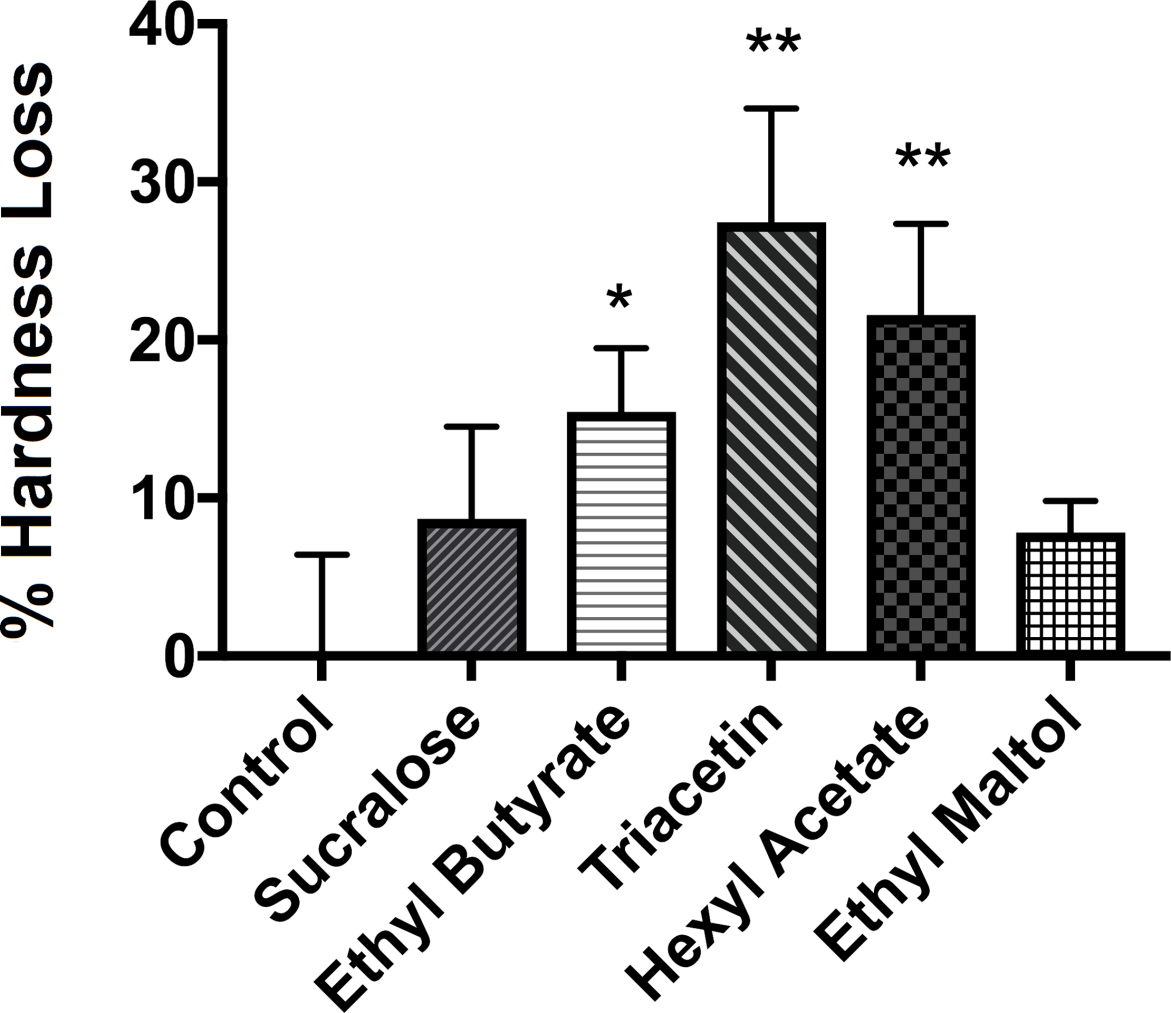
Enamel hardness loss after flavored e-liquid aerosol exposures. The hardness loss for control, sucralose, ethyl butyrate, triacetin, hexyl acetate and ethyl maltol were (0.01 ± 6.41, 8.67 ± 5.84, and 15.45 ± 4.02, 27.45 ± 7.19, 21.57 ± 5.76, and 7.80 ± 2.00) %, respectively (mean ± S.D.). Student t-tests were performed control vs. individual flavored e-liquid and statistical differences were indicated as: * = p<0.05 or ** = p<0.005.

To determine if flavors in e-liquids will increase demineralization of healthy enamel surface, the hardness of the surface was compared among enamel disks exposed to five different e-liquid aerosols (**Fig 6**). The baseline values were recorded by measuring hardness (three enamel disks per condition, five random locations per disk) prior to the aerosol exposure. The enamel disks were exposed using the standard protocol. After a 6 h incubation with *S*. *mutans*, the hardness of the disks was re-assessed by performing new indentations within 100 μm from the initial indents (prior to aerosol exposure). The percentage hardness loss (%) for control, sucralose, ethyl butyrate, triacetin, hexyl acetate, and ethyl maltol were (0.0004 ± 6.4, 8.6 ± 5.8, 15.4 ± 4.0 (p<0.05), 27.4 ± 7.1 (p<0.005), 21.5 ± 5.7 (p<0.005) and 7.8 ± 2.0) %, respectively.

### E-liquid base and flavors break down to smaller chemical by-products upon heating

Aerosols from control and five reference e-liquids were characterized using Gas Chromatography–Mass Spectrometry (GC-MS) (**Table 2**). Propylene glycol, nicotine, 2-propanol, diphenyl ether and nicotyrine were identified in aerosols generated from all six e-liquids. Generally, upon heating, e-liquids broke down to several alcohols, aromatic hydrocarbons, carboxylic acids, esters, aldehydes, ureas, carbonyl compounds, and ethers. Each flavor had at least one chemical by-product that was not present in the control aerosol (indicated by an asterisk). Ethyl maltol produced the most unique chemical by-products and sucralose aerosol had the least. **Table 2** also contains Signal-to-Noise (S/N) ratios which are based on Total Ion Chromatogram (TIC) intensity peak values. S/N ratio is important parameter for sensitivity and chromatography quality evaluation. The chemical by-products shown in **Table 2** are based on detectable peaks and suggested by the NIST Mass Spectral Library.

**Table 2:** GS-MS analyses of e-cigarette aerosols.

Group	CAS number	Name	S/N ratio
**Control**	57-55-6	Propylene Glycol	34.7
	54-11-5	Nicotine	53.7
	20324-32-7	2-Propanol, 1-(2-methoxy-1-methylethoxy)-	61.9
	101-84-8	Diphenyl ether	30.7
	487-19-4	Nicotyrine	17.2
	*Alcohol*		
	13588-28-8	1-Propanol, 2-(2-methoxypropoxy)-	51.8
	22104-79-6	2-Nonen-1-ol	7.62
	3944-36-3	2-Propanol, 1-(1-methylethoxy)-	4.49
	116-09-6	2-Propanone, 1-hydroxy-(Hydroxyacetone)	44.3
	*Aromatic hydrocarbon*		
	1014-60-4	Benzene, 1,3-bis(1,1-dimethylethyl)-	29.1
**Sucralose (C_12_H_19_Cl_3_O_8_)**	57-55-6	Propylene Glycol	34.1
	54-11-5	Nicotine	57.4
	20324-32-7	2-Propanol, 1-(2-methoxy-1-methylethoxy)-	67
	101-84-8	Diphenyl ether	56.9
	487-19-4	Nicotyrine	
	Alcohol		
	22104-79-6	*2-Nonen-1-ol	7.18
	*Carboxylic acid*		
	55536-71-5	*Di-tert-butyl 1,4-Dihydro-2,6-dimethyl-3,5-pyridinedicarboxylate	15.1
	*Aromatic hydrocarbon*		
	1014-60-4	*Benzene, 1,3-bis(1,1-dimethylethyl)-	28.3
**Ethyl Butyrate (C_6_H_12_O_2_)**	57-55-6	Propylene glycol	66.7
	54-11-5	Nicotine	56.8
	20324-32-7	2-Propanol, 1-(2-methoxy-1-methylethoxy)-	38.4
	101-84-8	Diphenyl ether	58.1
	487-19-4	Nicotyrine	27
	*Alcohol*		
	108-61-2	*1-Propanol, 2,2'-oxybis-	21.2
	104-76-7	*1-Hexanol, 2-ethyl-	9.04
	*Ester*		
	105-54-4	*Ethyl butyrate	96.1
	*Aromatic hydrocarbon*		
		*Benzenemethanol, α,α-dimethyl-	29.6
	*Aldehyde*		
	124-19-6	*Nonanal (C_9_H_18_O)	38.1
**Triacetin (C_9_H_14_O_6_)**	4254-14-2	Propylene glycol	68.2
	54-11-5	Nicotine	67
	20324-32-7	2-Propanol, 1-(2-methoxy-1-methylethoxy)-	43.9
	101-84-8	Diphenyl ether	55.8
	487-19-4	Nicotyrine	23.7
	*Alcohol*		
	116-09-6	*2-Propanone, 1-hydroxy-(C_3_H_6_O_2_)	38
	54305-61-2	*2-Butanol, 3,3'-oxybis-	35.1
	*Ester*		
	102-62-5	*Glycerol 1,2-diacetate	49.3
	*Aromatic hydrocarbon*		
	1014-60-4	*Benzene, 1,3-bis(1,1-dimethylethyl)-	15.5
**Hexyl Acetate (C_8_H_16_O_2_)**	57-55-6	Propylene glycol	34.5
	54-11-5	Nicotine	70.2
	20324-32-7	2-Propanol, 1-(2-methoxy-1-methylethoxy)-	45.4
	101-84-8	Diphenyl ether	64.3
	478-19-4	Nicotyrine	23.9
	*Alcohol*		
	111-27-3	*1-Hexanol	28.3
	*Ester*		
	142-92-7	*Hexyl acetate	90.1
	95-92-1	*Ethanedioic acid, diethyl ester	62.8
	*Aromatic hydrocarbon*		
	1014-60-4	*Benzene, 1,3-bis(1,1-dimethylethyl)-	35.2
	*Aldehyde*		
	124-19-6	*Nonanal (C_9_H_18_O)	54.7
	*Carboxylic acid*		
	26164-26-1	*Benzeneacetic acid, α-methoxy-, (S)- (C_6_H_5_CH(OCH_3_)CO_2_H)	6.13
	*Urea*		
	598-50-5	*N-Methylurea (CH_3_NHCONH_2_)	33.3
**Ethyl Maltol (C_7_H_8_O_3_)**	57-55-6	Propylene Glycol	32.2
	54-11-5	Nicotine	57.9
	20324-32-7	2-Propanol, 1-(2-methoxy-1-methylethoxy)-	60.2
	101-84-8	Diphenyl ether	32.8
	478-19-4	Nicotyrine	
	*Alcohol*		
	13588-28-8	*1-Propanol, 2-(2-methoxypropoxy)-	66.5
	54305-61-2	*2-Butanol, 3,3'-oxybis-	55
	4940-11-8	*Ethyl maltol	66.2
	*Aromatic hydrocarbon*		
	1014-60-4	*Benzene, 1,3-bis(1,1-dimethylethyl)-	49.1
	108-41-8	*Benzene, 1-chloro-3-methyl-	35.1
	95-49-8	*Benzene, 1-chloro-2-methyl-	43.8
	*Aldehyde*		
	124-19-6	*Nonanal (C_9_H_18_O)	24.3
	*Carbonyl compound*		
	1874-54-0	*Psicofuranine (C_11_H_15_N_5_O_5_)	29.3
	*Ether*		
	3386-87-6	*3,3'-(Ethylenedioxy) dipropionitrile (C_8_H_12_N_2_O_2_)	24.5

* = a unique chemical by-product that is not present in the control aerosol. These chemical by-products are based on detectable peaks and suggested by the NIST Mass Spectral Library.

### Sub-ohm e-cigarette aerosol contains metals

To identify types of metals in e-cigarette aerosol, Inductively Coupled Plasma with Optical Emission Spectrometry (ICP-OES) analyses were performed on 7.5 L (150 puffs) aerosol samples generated using the flavor-free reference e-liquid, a sub-ohm (0.2 Ω) heating element with a cotton-based wick. The presence of calcium (0.409 ± 0.002) mg/L, copper (0.011 ± 0.001) mg/L, iron (0.0051 ± 0.0003) mg/L, magnesium (0.017 ± 0.002) mg/L, and silicon (0.166 ± 0.005) mg/L was confirmed from the analysis of the aerosol. In contrast to previous findings, the new sub-ohm device did not emit lead or manganese [26]. The levels of cadmium (Cd), cobalt (Co), chromium (Cr), nickel (Ni), and palladium (Pd) were below the limit of ICP-OES detection. The results are summarized in **Table 3** with the National Institute of Occupational Safety and Health (NIOSH) Daily Exposure Limit information.

**Table 3:** Metals in e-cigarette aerosol.

	Calcium	Copper	Iron	Magnesium	Silicon
**Sub-ohm:** **0.2 Ω** **49.2 W** **150 puffs (mg/L)** ^**a**^	0.409 ± 0.002	0.011 ± 0.001	0.0051 ± 0.0003	0.017 ± 0.002	0.166 ± 0.005
**NIOSH:** **Daily exposure limits (mg/L)**	5	1	5	15	5

^**a**^ The concentrations of the metals (mg/L) are shown as (mean ± S.D.).

## Discussion

Dental caries is a complex disease with many etiological factors including host genetics, oral microbiome, immune system, diet, oral hygiene, salivary function, community water fluoridation, and access to quality dental care [56]. The direct correlation between diet, especially the quantity of sucrose intake, and dental caries incidence has been intensely researched and supported by many studies [56, 57]. In addition to the quantity of sucrose intake, how it is delivered (e.g., sugar mixed in acidic beverages), how often it is delivered (e.g., sipping sugary beverage over an extended time), and how long the sucrose is in contact with the hard tissue surface (e.g., hard and sticky candies can lead to longer sucrose exposure in the oral cavity) can further increase the risk of initiation, progress, and severity of dental caries [58, 59]. Although there is strong scientific evidence showing that a diet high in sucrose is the most important factor in caries development, the similar sugary and acidic flavors (e.g., saccharides, esters, acids, and aldehydes) found in e-liquids have not been studied to the same extent. Since the main route of intake and sensory perception of foods (mastication via oral cavity to gastrointestinal tract) and e-liquids (inhalation via oral cavity to respiratory tract) are not exactly the same, caution should be taken when flavors of e-liquids are compared to actual foods and beverages. However, identification of specific flavors that increase cariogenic potential will facilitate the development of the oral health risk assessment of e-cigarette use and provide scientific evidence that there may be unintended consequences of using e-cigarettes.

In this study, *Streptococcus mutans* was exposed to flavored e-liquid aerosols to identify specific flavors and chemical by-products that may increase tooth surface damage. Through a combination of pure reference e-liquid and GC-MS based analysis, we discovered that ethyl butyrate, triacetin and hexyl acetate and their respective chemical by-products increase cariogenic potential. Ethyl butyrate, hexyl acetate, and triacetin are different types of esters. Ethyl butyrate possesses a strong pineapple scent and is naturally produced in many fruits [59]. Interestingly, several oral bacteria including *Streptococcus salivarius* and *Lactococcus lactis* actively produce ethyl butyrate [60]. This indicates *S*. *mutans* is frequently exposed to ethyl butyrate in oral biofilm and at minimum tolerates or as suggested by the results, can enhance biofilm development in response. Hexyl acetate has not been studied in the context of oral biofilm, dental caries or *S*. *mutans*. However, *Streptococcus*, *Actinomyces*, and *Lactobacillus* via classic glycolysis metabolize carbohydrates to acetate in oral biofilm [61]. With acetate being one of final principle products formed by biofilm bacteria [62], it is expected that *S*. *mutans* is able to thrive in an acetate-rich environment. Triacetin is found in fruits and cigarette filters and has a “velvety” or “smoky” flavor. Triacetin is mainly used as a food additive, humectant, plasticizer, and anti-knocking agent but not much is known in the context of oral biology [62]. It is, however, well known that *S*. *mutans* has esterase activities that degrade monomers in dental restorative materials such as resin composites and adhesives [63]. Based on these data, it is proposed that esters in e-liquid flavors provide an additional food source for *S*. *mutans* to flourish in oral biofilm environment. However, further metabolomic analysis should be performed to validate the findings described here.

Further examination of the biofilm assays, mechanical testing (adhesion force and hardness measurement), and SEM images show complex surface changes and biological responses upon e-cigarette aerosol exposure. The results demonstrate that an e-cigarette produces viscous aerosols which cover enamel surfaces. Surface characteristics such as surface roughness, tackiness, charge, and energy have a significant impact on how bacterial cells adhere to surfaces and subsequently form biofilm [64]. The data shown in this study are consistent with previous studies that adhesion of *S*. *mutans* to surfaces can be influenced by several factors (e.g., presence of acquired pellicles, salivary proteins, specific glucan synthesis, cell surface proteins, other oral bacteria and availability of sucrose) [65–67]. This study confirms that *S*. *mutans*-to-surface interactions can be altered by e-cigarette aerosols. Previous studies have shown that *S*. *mutans* are able to exploit the initial attachment to tooth surfaces by secreting Extracellular Polymeric Substances (EPS) [68–70]. EPS allow *S*. *mutans* to encapsulate itself on the surface, start multiplying in number and eventually forming biofilm [71, 72]. *S*. *mutans* in biofilm can rapidly metabolize carbohydrates into lactic acid, creating locally a low pH, leading to demineralization of enamel surface [56]. Our SEM and micro-indentation hardness analyses suggest that *S*. *mutans* attach to the e-cigarette exposed surface, metabolize e-liquid base and flavors to secrete EPS and rapidly form biofilm which demineralize the e-cigarette exposed enamel surfaces. Demineralization of enamel is of great importance to oral health because the demineralization is the first step of dental caries development.

*S*. *mutans* have evolved to survive in challenging environment by developing remarkable metabolic flexibility [73]. In this study, levels of 12 metal ions in e-cigarette aerosol have been measured. At a high concentration, metals can be toxic to bacteria and humans, though, at physiological levels, metal ions may serve as nutrients required for many important biological processes [73]. Oral bacteria including *S*. *mutans* require metal ions (e.g., copper, iron, and magnesium) as a co-factor to activate essential enzymes [73]. Pathogenic bacteria also have evolved numerous mechanisms for essential metal uptake to circumvent the host’s immune system [73]. By ICP-OES analyses, the following metals in e-cigarette aerosol were identified: calcium, copper, iron, magnesium, and silicon. Calcium, iron and copper ions are well-known modulators in biofilm formation and enamel remineralization / demineralization processes [74–76]. *S*. *mutans* is known to have a nutritional requirement for magnesium [77]. Our data suggest that the level of metals is well tolerated by *S*. *mutans* following the e-cigarette aerosol exposure as described in the Study Design.

The non-linear correlation between the absorbance data and the hardness loss data indicate that the mechanism of bacterial growth and enamel demineralization is complex. Therefore, in addition to the bacteria-initiated damage to the enamel surface, the chemical by-products may influence the hardness of the surface directly. Previous studies have demonstrated acidic drinks, citrus suckling behavior, and bulimia can lead to enamel surface damage directly [78].

Using AFM, it was found that the adhesive forces between *S*. *mutans* and enamel surface increases as a function of number of puffs. It was also found that with 10 puffs, aerosol droplets were evenly distributed which was verified by the force measurement and light microscope images. However, as the number of puffs increased, aerosol droplets started to aggregate, which may explain the larger variation recorded from 150 puff samples. Whether the local accumulation of droplets can be recapitulated in *in-vivo* system and whether it has important biological implications remain to be seen.

Ethyl maltol has a distinctive fragrance that resembles cotton candy. It is one of the strongest fragrances tested in this study and remains a popular additive among commercial e-liquids. It was unexpected to find that ethyl maltol in e-liquids acted as a potent antimicrobial agent against *S*. *mutans*. Although ethyl maltol was not investigated as a therapeutic antibiotic, Schved *et al*. have shown that ethyl maltol destabilizes the outer cell membrane of *E*. *coli* by chelating Mg^2+^ and/or Ca^2+^ in a pH dependent manner [79]. Thus, it is plausible that ethyl maltol may interfere with *S*. *mutans* cell membrane integrity in a similar fashion.

There are several limitations to this study. The primary limitation of this study is that the oral microbiome is a complex network of several hundred bacteria species. In this study, the biological responses were characterized only from one organism, *Streptococcus mutans*, a major cariogenic bacterium in oral cavity. Since other oral bacteria have different nutritional requirements and can tolerate different levels of environmental challenges such as pH changes or chemical exposures, they may respond differently to the flavored e-liquids tested here. Although the reference e-liquids intentionally used only one flavor per e-liquid, commercial e-liquids contain several additives, including sucrose, sugar substitutes and acids, some at much higher concentration [27–29, 80]. This suggests that the actual damage to the tooth enamel surface may vary with the constituents present in e-liquids, and could be higher or lower than measured in this study. Since e-liquid undergoes thermal degradation when aerosolized, the concentration of flavors in the resulting aerosol may be different from the concentration of flavors in the starting e-liquid. For example, Rosbrook *et al*. reported that the amount of sucralose in aerosols can be altered by e-cigarette delivery systems such as wick design and size of mouthpiece, and interestingly, not necessarily by voltage or resistance of the metal heating element [81]. This suggests that (1) the concentration of flavors in aerosols may be difficult to predict without actual experimental quantification, and (2) the concentration of flavors in aerosols depends on the constituents of the starting e-liquid as well as the design of the device. Recently, Krusemann *et al*. systematically classified commercial e-liquids into a comprehensive chart AKA “E-Liquid Flavor Wheel” [5]. The flavor wheel suggests there may be other flavors (e.g., alcohol, honey, or vanilla) which could damage tooth enamel as well. A limitation of the study is that the bacterial culturing protocol could not directly incorporate human saliva (for its buffering ability to counteract low pH challenges) into the *in-vitro* experimental design. To counteract this limitation, *S*. *mutans* were grown in buffered media. Although ICP-OES data suggested that the level of metals was below the National Institute of Occupational Safety and Health (NIOSH) Daily Exposure Limit (**Table 3**), the experimental conditions were ideal and conservative as possible. This was intentional to improve transparency and reproducibility of the research methods described in this study. *In-vivo* metal levels may vary depending on devices, e-liquids, flavors, puff and inter-puff durations, heating element resistance, wattage of the system and user’s behavior patterns (e.g., compliance to the manufacturer’s instruction). It is possible that the chronic metal exposure even at a low to moderate levels may lead to unintended microbial dysbiosis causing negative health consequences [82–85]. Finally, humans’ flavor perception is a complex neurophysiological phenomenon. Perceptions of the flavors of foods or beverages reflect information received from multiple sensory afferents, including gustatory (taste), olfactory (smell), and somatosensory fibers [86]. As such, some flavors in commercial e-liquids are added to enhance positive gustatory (e.g., sucralose) input or olfactory sensation (e.g. ethyl maltol) or a combination of both. Although descriptions of commercial e-liquids may resemble actual foods or beverages, the similarity between two products is mainly achieved by manipulating the olfaction of the users through inhalation. A fraction of aerosols will be dissolved by saliva and transported to the sweet taste receptors mainly located on the tongue and palate of oral cavity. In this study, we primarily focused on bacteria-induced cariogenicity from certain flavors, chemical by-products and viscosity of glycerin. However, the impact of flavors in e-cigarette products on human health may be more significant than previously described.

Despite these limitations, this study provides insight into potential unintended negative consequences of vaping on oral health, specifically teeth. Although tooth enamel is the hardest mineralized tissue in human body, once damaged beyond salivary buffering capacity, it has no way of regenerating itself. Based on the results and aforementioned limitations, there are at least two immediate needs to advance this work: (1) clinical investigations should be performed to confirm and translate the data shown here, and (2) case studies based on clinical observations from oral health providers will greatly enhance our understanding of the real cost of e-cigarette on oral health.

## Conclusions

A novel finding of this study is that certain e-liquid ingredients interact with hard tissues of the oral cavity in such a way that resembles high-sucrose candies and acidic drinks that adversely affect teeth. This is an important finding that suggests the complexity of e-cigarettes on human health goes beyond respiratory and cardiac systems and may have significant implications on oral health. It is a common perception among e-cigarette users that vaping is less harmful or is without health risk. Though it is acknowledged here that e-cigarette aerosols contain less harmful and potentially harmful constituents compared to combustible tobacco products, the data suggests e-cigarettes produce viscous aerosols that change surface characteristics and have biological consequences. Viscous e-liquids made from propylene glycol and glycerin, along with sweet flavors facilitate attachment and provide additional food source which pathogenic oral bacteria such as *S*. *mutans* prefer. Youth and young adults are a uniquely vulnerable population to dental caries due to their high-sucrose diet and poor to minimal oral hygiene practice [87–90]. This study suggests that flavored e-cigarette products negatively affect teeth and pose potential oral health risk. These two facts advocate that there is an urgent need to further research e-cigarettes, e-liquids and flavors in the context of human health and disease.

## References

[1] Singh T, Arrazola RA, Corey CG, Husten CG, Neff LJ, Homa DM, et al Tobacco Use Among Middle and High School Students—United States, 2011–2015. MMWR Morb Mortal Wkly Rep. 2016;65(14):361–7. doi: 10.15585/mmwr.mm6514a1 . 27077789 10.15585/mmwr.mm6514a1

[2] Jamal A, Gentzke A, Hu SS, Cullen KA, Apelberg BJ, Homa DM, et al Tobacco Use Among Middle and High School Students—United States, 2011–2016. MMWR Morb Mortal Wkly Rep. 2017;66(23):597–603. doi: 10.15585/mmwr.mm6623a1 ; PubMed Central PMCID: PMCPMC5657845. 28617771 10.15585/mmwr.mm6623a1 PMC5657845

[3] Lindblom EN. Effectively Regulating E-Cigarettes and Their Advertising—And the First Amendment. Food Drug Law J. 2015;70(1):55–92. . 26292472

[4] Pearson JL, Richardson A, Niaura RS, Vallone DM, Abrams DB. e-Cigarette awareness, use, and harm perceptions in US adults. Am J Public Health. 2012;102(9):1758–66. 10.2105/AJPH.2011.300526 ; PubMed Central PMCID: PMCPMC3474361. 22813087 PMC3474361

[5] Krusemann EJZ, Boesveldt S, de Graaf K, Talhout R. An E-liquid Flavor Wheel: A Shared Vocabulary based on Systematically Reviewing E-liquid Flavor Classifications in Literature. Nicotine Tob Res. 2018 10.1093/ntr/nty101 . 29788484 PMC6751518

[6] Ambrose BK, Day HR, Rostron B, Conway KP, Borek N, Hyland A, et al Flavored Tobacco Product Use Among US Youth Aged 12–17 Years, 2013–2014. JAMA. 2015;314(17):1871–3. 10.1001/jama.2015.13802 . 26502219 PMC6467270

[7] Singh T, Kennedy S, Marynak K, Persoskie A, Melstrom P, King BA. Characteristics of Electronic Cigarette Use Among Middle and High School Students—United States, 2015. MMWR Morb Mortal Wkly Rep. 2016;65(5051):1425–9. doi: 10.15585/mmwr.mm655051a2 . 28033310 10.15585/mmwr.mm655051a2

[8] Soneji SS, Sung HY, Primack BA, Pierce JP, Sargent JD. Quantifying population-level health benefits and harms of e-cigarette use in the United States. PLoS One. 2018;13(3):e0193328 10.1371/journal.pone.0193328 ; PubMed Central PMCID: PMCPMC5851558. 29538396 PMC5851558

[9] Schneider S, Diehl K. Vaping as a Catalyst for Smoking? An Initial Model on the Initiation of Electronic Cigarette Use and the Transition to Tobacco Smoking Among Adolescents. Nicotine Tob Res. 2016;18(5):647–53. 10.1093/ntr/ntv193 . 26386472

[10] Soneji S, Barrington-Trimis JL, Wills TA, Leventhal AM, Unger JB, Gibson LA, et al Association Between Initial Use of e-Cigarettes and Subsequent Cigarette Smoking Among Adolescents and Young Adults: A Systematic Review and Meta-analysis. JAMA Pediatr. 2017;171(8):788–97. 10.1001/jamapediatrics.2017.1488 ; PubMed Central PMCID: PMCPMC5656237. 28654986 PMC5656237

[11] H.R. 1256-111th Congress: Family Smoking Prevention and Tobacco Control Act In: HR 1256: GovTrack.us (database of federal legislation); 2009.

[12] European Comission. The Tobacco Products Directive (2014/40/EU). 2014 http://ec.europa.eu/health/tobacco/docs/dir_201440_en.pdf. (accessed Aug. 2, 2018)

[13] Villanti AC, Johnson AL, Ambrose BK, Cummings KM, Stanton CA, Rose SW, et al Flavored Tobacco Product Use in Youth and Adults: Findings From the First Wave of the PATH Study (2013–2014). Am J Prev Med. 2017;53(2):139–51. 10.1016/j.amepre.2017.01.026 ; PubMed Central PMCID: PMCPMC5522636. 28318902 PMC5522636

[14] Food, Drug Administration HHS. Deeming Tobacco Products To Be Subject to the Federal Food, Drug, and Cosmetic Act, as Amended by the Family Smoking Prevention and Tobacco Control Act; Restrictions on the Sale and Distribution of Tobacco Products and Required Warning Statements for Tobacco Products. Final rule. Fed Regist. 2016;81(90):28973–9106. . 27192730

[15] Kowitt SD, Meernik C, Baker HM, Osman A, Huang LL, Goldstein AO. Perceptions and Experiences with Flavored Non-Menthol Tobacco Products: A Systematic Review of Qualitative Studies. Int J Environ Res Public Health. 2017;14(4). 10.3390/ijerph14040338 ; PubMed Central PMCID: PMCPMC5409539. 28333107 PMC5409539

[16] Zhu SH, Sun JY, Bonnevie E, Cummins SE, Gamst A, Yin L, et al Four hundred and sixty brands of e-cigarettes and counting: implications for product regulation. Tob Control. 2014;23 Suppl 3:iii3–9. 10.1136/tobaccocontrol-2014-051670 ; PubMed Central PMCID: PMCPMC4078673. 24935895 PMC4078673

[17] Goniewicz ML, Kuma T, Gawron M, Knysak J, Kosmider L. Nicotine levels in electronic cigarettes. Nicotine Tob Res. 2013;15(1):158–66. 10.1093/ntr/nts103 . 22529223

[18] Goniewicz ML, Knysak J, Gawron M, Kosmider L, Sobczak A, Kurek J, et al Levels of selected carcinogens and toxicants in vapour from electronic cigarettes. Tob Control. 2014;23(2):133–9. 10.1136/tobaccocontrol-2012-050859 ; PubMed Central PMCID: PMCPMC4154473. 23467656 PMC4154473

[19] Hutzler C, Paschke M, Kruschinski S, Henkler F, Hahn J, Luch A. Chemical hazards present in liquids and vapors of electronic cigarettes. Arch Toxicol. 2014;88(7):1295–308. 10.1007/s00204-014-1294-7 . 24958024

[20] Trehy ML, Ye W, Hadwiger ME, Moore TW, Allgire JF, Woodruff JT, et al Analysis of Electronic Cigarette Cartridges, Refill Solutions, and Smoke for Nicotine and Nicotine Related Impurities. Journal of Liquid Chromatography & Related Technologies. 2011;34(14):1442–58. 10.1080/10826076.2011.572213 PubMed PMID: WOS:000296230900012.

[21] Cobb NK, Byron MJ, Abrams DB, Shields PG. Novel nicotine delivery systems and public health: the rise of the "e-cigarette". Am J Public Health. 2010;100(12):2340–2. Epub 2010/11/12. 10.2105/AJPH.2010.199281 ; PubMed Central PMCID: PMC2978165. 21068414 PMC2978165

[22] Cameron JM, Howell DN, White JR, Andrenyak DM, Layton ME, Roll JM. Variable and potentially fatal amounts of nicotine in e-cigarette nicotine solutions. Tob Control. 2014;23(1):77–8. Epub 2013/02/15. 10.1136/tobaccocontrol-2012-050604 . 23407110

[23] Cheah NP, Chong NW, Tan J, Morsed FA, Yee SK. Electronic nicotine delivery systems: regulatory and safety challenges: Singapore perspective. Tob Control. 2014;23(2):119–25. Epub 2012/12/04. 10.1136/tobaccocontrol-2012-050483 . 23204074

[24] Etter JF, Zather E, Svensson S. Analysis of refill liquids for electronic cigarettes. Addiction. 2013;108(9):1671–9. Epub 2013/05/25. 10.1111/add.12235 . 23701634

[25] Hadwiger ME, Trehy ML, Ye W, Moore T, Allgire J, Westenberger B. Identification of amino-tadalafil and rimonabant in electronic cigarette products using high pressure liquid chromatography with diode array and tandem mass spectrometric detection. J Chromatogr A. 2010;1217(48):7547–55. Epub 2010/10/29. 10.1016/j.chroma.2010.10.018 . 20980012

[26] Kim JJ, Sabatelli N, Tutak W, Giuseppetti A, Frukhtbeyn S, Shaffer I, et al Universal electronic-cigarette test: physiochemical characterization of reference e-liquid. Tob Induc Dis. 2017;15:14 10.1186/s12971-017-0119-x ; PubMed Central PMCID: PMCPMC5314484. 28239329 PMC5314484

[27] Soussy S, El-Hellani A, Baalbaki R, Salman R, Shihadeh A, Saliba NA. Detection of 5-hydroxymethylfurfural and furfural in the aerosol of electronic cigarettes. Tob Control. 2016;25(Suppl 2):ii88–ii93. 10.1136/tobaccocontrol-2016-053220 . 27798321

[28] Tierney PA, Karpinski CD, Brown JE, Luo W, Pankow JF. Flavour chemicals in electronic cigarette fluids. Tob Control. 2016;25(e1):e10–5. 10.1136/tobaccocontrol-2014-052175 ; PubMed Central PMCID: PMCPMC4853541. 25877377 PMC4853541

[29] https://www.nudenicotine.com/product/sucralose-solutions-5-15/ (accessed Aug. 2, 2018).

[30] Frostell G, Keyes PH, Larson RH. Effect of various sugars and sugar substitutes on dental caries in hamsters and rats. J Nutr. 1967;93(1):65–76. 10.1093/jn/93.1.65 . 6053761

[31] Keyes PH. Dental caries in the Syrian hamster. VI. Minimal dental caries activity in animals fed presumably cariogenic rations. J Dent Res. 1954;33(6):830–41. 10.1177/00220345540330061101 . 13211876

[32] Huilgol P, Bhatt SP, Biligowda N, Wright NC, Wells JM. Association of e-cigarette use with oral health: a population-based cross-sectional questionnaire study. J Public Health (Oxf). 2018 10.1093/pubmed/fdy082 . 29788415 PMC6636695

[33] Berg JH. The marketplace for new caries management products: dental caries detection and caries management by risk assessment. BMC Oral Health. 2006;6 Suppl 1:S6 10.1186/1472-6831-6-S1-S6 ; PubMed Central PMCID: PMCPMC2147594. 16934123 PMC2147594

[34] Featherstone JD. The science and practice of caries prevention. J Am Dent Assoc. 2000;131(7):887–99. . 10916327 10.14219/jada.archive.2000.0307

[35] Zero DT. Dental caries process. Dent Clin North Am. 1999;43(4):635–64. . 10553248

[36] Leung V, Dufour D, Levesque CM. Death and survival in Streptococcus mutans: differing outcomes of a quorum-sensing signaling peptide. Front Microbiol. 2015;6:1176 10.3389/fmicb.2015.01176 ; PubMed Central PMCID: PMCPMC4615949. 26557114 PMC4615949

[37] Lemos JA, Burne RA. A model of efficiency: stress tolerance by Streptococcus mutans. Microbiology. 2008;154(Pt 11):3247–55. 10.1099/mic.0.2008/023770-0 ; PubMed Central PMCID: PMCPMC2627771. 18957579 PMC2627771

[38] Smith EG, Spatafora GA. Gene regulation in S. mutans: complex control in a complex environment. J Dent Res. 2012;91(2):133–41. 10.1177/0022034511415415 . 21743034

[39] Schilling KM, Bowen WH. Glucans synthesized in situ in experimental salivary pellicle function as specific binding sites for Streptococcus mutans. Infect Immun. 1992;60(1):284–95. ; PubMed Central PMCID: PMCPMC257534. 1530843 10.1128/iai.60.1.284-295.1992 PMC257534

[40] Bowen WH, Koo H. Biology of Streptococcus mutans-derived glucosyltransferases: role in extracellular matrix formation of cariogenic biofilms. Caries Res. 2011;45(1):69–86. 10.1159/000324598 ; PubMed Central PMCID: PMCPMC3068567. 21346355 PMC3068567

[41] Tatevossian A. Facts and artefacts in research on human dental plaque fluid. J Dent Res. 1990;69(6):1309–15. 10.1177/00220345900690061801 . 2191981

[42] Hayacibara MF, Koo H, Vacca-Smith AM, Kopec LK, Scott-Anne K, Cury JA, et al The influence of mutanase and dextranase on the production and structure of glucans synthesized by streptococcal glucosyltransferases. Carbohydr Res. 2004;339(12):2127–37. 10.1016/j.carres.2004.05.031 . 15280057

[43] Flemming HC, Wingender J. The biofilm matrix. Nat Rev Microbiol. 2010;8(9):623–33. 10.1038/nrmicro2415 . 20676145

[44] Wilson RF, Ashley FP. Relationships between the biochemical composition of both free smooth surface and approximal plaque and salivary composition and a 24-hour retrospective dietary history of sugar intake in adolescents. Caries Res. 1990;24(3):203–10. 10.1159/000261266 . 2364406

[45] Featherstone JD. The continuum of dental caries—evidence for a dynamic disease process. J Dent Res. 2004;83 Spec No C:C39–42. . 15286120 10.1177/154405910408301s08

[46] Behar RZ, Hua M, Talbot P. Puffing topography and nicotine intake of electronic cigarette users. PLoS One. 2015;10(2):e0117222 10.1371/journal.pone.0117222 ; PubMed Central PMCID: PMCPMC4321841. 25664463 PMC4321841

[47] Spindle TR, Breland AB, Karaoghlanian NV, Shihadeh AL, Eissenberg T. Preliminary results of an examination of electronic cigarette user puff topography: the effect of a mouthpiece-based topography measurement device on plasma nicotine and subjective effects. Nicotine Tob Res. 2015;17(2):142–9. 10.1093/ntr/ntu186 ; PubMed Central PMCID: PMCPMC4838000. 25239957 PMC4838000

[48] Etter JF. A longitudinal study of cotinine in long-term daily users of e-cigarettes. Drug Alcohol Depend. 2016;160:218–21. 10.1016/j.drugalcdep.2016.01.003 . 26804899

[49] Li Q, Zhan Y, Wang L, Leischow SJ, Zeng DD. Analysis of symptoms and their potential associations with e-liquids' components: a social media study. BMC Public Health. 2016;16:674 10.1186/s12889-016-3326-0 ; PubMed Central PMCID: PMCPMC4967297. 27475060 PMC4967297

[50] http://vaping360.com/pg-vs-vg-what-is-the-difference-and-what-should-i-use/ (accessed Aug. 2, 2018).

[51] Lippert F, Lynch RJ. Comparison of Knoop and Vickers surface microhardness and transverse microradiography for the study of early caries lesion formation in human and bovine enamel. Arch Oral Biol. 2014;59(7):704–10. 10.1016/j.archoralbio.2014.04.005 . 24798979

[52] Craig RG, Gehring PE, Peyton FA. Relation of structure to the microhardness of human dentin. J Dent Res. 1959;38(3):624–30. 10.1177/00220345590380032701 . 13654615

[53] O'Toole GA. Microtiter dish biofilm formation assay. J Vis Exp. 2011;(47). 10.3791/2437 ; PubMed Central PMCID: PMCPMC3182663. 21307833 PMC3182663

[54] Lopez Perez D, Baker PJ, Pintar AL, Sun J, Lin NJ, Lin-Gibson S. Experimental and statistical methods to evaluate antibacterial activity of a quaternary pyridinium salt on planktonic, biofilm-forming, and biofilm states. Biofouling. 2017;33(3):222–34. 10.1080/08927014.2017.1286476 . 28270052

[55] Kilian M, Chapple IL, Hannig M, Marsh PD, Meuric V, Pedersen AM, et al The oral microbiome—an update for oral healthcare professionals. Br Dent J. 2016;221(10):657–66. 10.1038/sj.bdj.2016.865 . 27857087

[56] Pitts NB, Zero DT, Marsh PD, Ekstrand K, Weintraub JA, Ramos-Gomez F, et al Dental caries. Nat Rev Dis Primers. 2017;3:17030 10.1038/nrdp.2017.30 . 28540937

[57] Sheiham A, James WP. Diet and Dental Caries: The Pivotal Role of Free Sugars Reemphasized. J Dent Res. 2015;94(10):1341–7. 10.1177/0022034515590377 . 26261186

[58] Anderson CA, Curzon ME, Van Loveren C, Tatsi C, Duggal MS. Sucrose and dental caries: a review of the evidence. Obes Rev. 2009;10 Suppl 1:41–54. 10.1111/j.1467-789X.2008.00564.x . 19207535

[59] Moynihan P, Petersen PE. Diet, nutrition and the prevention of dental diseases. Public Health Nutr. 2004;7(1A):201–26. . 14972061 10.1079/phn2003589

[60] Liu SQ, Holland R, Crow VL. Ethyl butanoate formation by dairy lactic acid bacteria. International Dairy Journal. 1998;8(7):651–7. 10.1016/S0958-6946(98)00100-9 PubMed PMID: WOS:000077404700008.

[61] Sasaki K, Suzuki O, Takahashi N, Stashenko P. Interface oral health science 2011: proceedings of the 4th International Symposium for Interface Oral Health Science, Held in Sendai, Japan, Between March 7 and 8, 2011 and the Harvard-Forsyth-Tohoku Research Workshop, Held in Cambridge, USA, Between January 6 and 7, 2011. Tokyo; New York: Springer; 2012. xx, 422 pages p.

[62] Kim JN, Ahn SJ, Burne RA. Genetics and Physiology of Acetate Metabolism by the Pta-Ack Pathway of Streptococcus mutans. Appl Environ Microbiol. 2015;81(15):5015–25. 10.1128/AEM.01160-15 ; PubMed Central PMCID: PMCPMC4495203. 25979891 PMC4495203

[63] Bourbia M, Ma D, Cvitkovitch DG, Santerre JP, Finer Y. Cariogenic bacteria degrade dental resin composites and adhesives. J Dent Res. 2013;92(11):989–94. 10.1177/0022034513504436 ; PubMed Central PMCID: PMCPMC3797536. 24026951 PMC3797536

[64] Tuson HH, Weibel DB. Bacteria-surface interactions. Soft Matter. 2013;9(18):4368–80. 10.1039/C3SM27705D ; PubMed Central PMCID: PMCPMC3733390. 23930134 PMC3733390

[65] Orstavik D, Kraus FW, Henshaw LC. In vitro attachment of streptococci to the tooth surface. Infect Immun. 1974;9(5):794–800. ; PubMed Central PMCID: PMCPMC414887. 4856824 10.1128/iai.9.5.794-800.1974 PMC414887

[66] Diaz-Garrido N, Lozano C, Giacaman RA. Frequency of sucrose exposure on the cariogenicity of a biofilm-caries model. Eur J Dent. 2016;10(3):345–50. 10.4103/1305-7456.184163 ; PubMed Central PMCID: PMCPMC4926586. 27403051 PMC4926586

[67] Matsumoto-Nakano M. Role of Streptococcus mutans surface proteins for biofilm formation. Jpn Dent Sci Rev. 2018;54(1):22–9. 10.1016/j.jdsr.2017.08.002 ; PubMed Central PMCID: PMCPMC5884221. 29628998 PMC5884221

[68] Guggenheim B. Extracellular polysaccharides and microbial plaque. Int Dent J. 1970;20(4):657–78. . 5276615

[69] Hamada S, Slade HD. Biology, immunology, and cariogenicity of Streptococcus mutans. Microbiol Rev. 1980;44(2):331–84. ; PubMed Central PMCID: PMCPMC373181. 6446023 10.1128/mr.44.2.331-384.1980 PMC373181

[70] Loesche WJ. Role of Streptococcus mutans in human dental decay. Microbiol Rev. 1986;50(4):353–80. ; PubMed Central PMCID: PMCPMC373078. 3540569 10.1128/mr.50.4.353-380.1986 PMC373078

[71] Banas JA, Vickerman MM. Glucan-binding proteins of the oral streptococci. Crit Rev Oral Biol Med. 2003;14(2):89–99. . 12764072 10.1177/154411130301400203

[72] Sutherland IW. Biotechnology of microbial exopolysaccharides Cambridge; New York: Cambridge University Press; 1990 viii, 163 p. p.

[73] Passalacqua KD, Charbonneau ME, O'Riordan MX. Bacterial Metabolism Shapes the Host-Pathogen Interface. Microbiol Spectr. 2016;4(3). 10.1128/microbiolspec.VMBF-0027-2015 ; PubMed Central PMCID: PMCPMC4922512. 27337445 PMC4922512

[74] Leitao TJ, Cury JA, Tenuta LMA. Kinetics of calcium binding to dental biofilm bacteria. PLoS One. 2018;13(1):e0191284 10.1371/journal.pone.0191284 ; PubMed Central PMCID: PMCPMC5791987. 29385163 PMC5791987

[75] Garcia SS, Du Q, Wu H. Streptococcus mutans copper chaperone, CopZ, is critical for biofilm formation and competitiveness. Mol Oral Microbiol. 2016;31(6):515–25. 10.1111/omi.12150 ; PubMed Central PMCID: PMCPMC5123798. 27753272 PMC5123798

[76] Berlutti F, Ajello M, Bosso P, Morea C, Petrucca A, Antonini G, et al Both lactoferrin and iron influence aggregation and biofilm formation in Streptococcus mutans. Biometals. 2004;17(3):271–8. . 15222477 10.1023/b:biom.0000027704.53859.d3

[77] Aranha H, Strachan RC, Arceneaux JE, Byers BR. Effect of trace metals on growth of Streptococcus mutans in a teflon chemostat. Infect Immun. 1982;35(2):456–60. ; PubMed Central PMCID: PMCPMC351061. 7035364 10.1128/iai.35.2.456-460.1982 PMC351061

[78] Spear F. A patient with severe wear on the anterior teeth and minimal wear on the posterior teeth. J Am Dent Assoc. 2008;139(10):1399–403. . 18832276 10.14219/jada.archive.2008.0052

[79] Schved F, Pierson MD, Juven BJ. Sensitization of Escherichia coli to nisin by maltol and ethyl maltol. Lett Appl Microbiol. 1996;22(3):189–91. . 8852344 10.1111/j.1472-765x.1996.tb01139.x

[80] http://e-liquid-recipes.com/ (accessed Aug. 2, 2018).

[81] Rosbrook K, Erythropel HC, DeWinter TM, Falinski M, O'Malley S, Krishnan-Sarin S, et al The effect of sucralose on flavor sweetness in electronic cigarettes varies between delivery devices. PLoS One. 2017;12(10):e0185334 10.1371/journal.pone.0185334 ; PubMed Central PMCID: PMCPMC5624589. 28968411 PMC5624589

[82] Gaetke LM, Chow CK. Copper toxicity, oxidative stress, and antioxidant nutrients. Toxicology. 2003;189(1–2):147–63. . 12821289 10.1016/s0300-483x(03)00159-8

[83] Rosenfeld CS. Gut Dysbiosis in Animals Due to Environmental Chemical Exposures. Front Cell Infect Microbiol. 2017;7:396 10.3389/fcimb.2017.00396 ; PubMed Central PMCID: PMCPMC5596107. 28936425 PMC5596107

[84] Zhou J, Jiang N, Wang Z, Li L, Zhang J, Ma R, et al Influences of pH and Iron Concentration on the Salivary Microbiome in Individual Humans with and without Caries. Appl Environ Microbiol. 2017;83(4). 10.1128/AEM.02412-16 ; PubMed Central PMCID: PMCPMC5288818. 27940544 PMC5288818

[85] Pietroiusti A, Magrini A, Campagnolo L. New frontiers in nanotoxicology: Gut microbiota/microbiome-mediated effects of engineered nanomaterials. Toxicol Appl Pharmacol. 2016;299:90–5. 10.1016/j.taap.2015.12.017 . 26723910

[86] Small DM, Prescott J. Odor/taste integration and the perception of flavor. Exp Brain Res. 2005;166(3–4):345–57. 10.1007/s00221-005-2376-9 . 16028032

[87] Peres MA, Sheiham A, Liu P, Demarco FF, Silva AE, Assuncao MC, et al Sugar Consumption and Changes in Dental Caries from Childhood to Adolescence. J Dent Res. 2016;95(4):388–94. 10.1177/0022034515625907 . 26758380

[88] Rugg-Gunn AJ, Hackett AF, Appleton DR, Jenkins GN, Eastoe JE. Relationship between dietary habits and caries increment assessed over two years in 405 English adolescent school children. Arch Oral Biol. 1984;29(12):983–92. . 6598368 10.1016/0003-9969(84)90145-6

[89] Rosinger A, Herrick K, Gahche J, Park S. Sugar-sweetened Beverage Consumption Among U.S. Youth, 2011–2014. NCHS Data Brief. 2017;(271):1–8. . 28135184

[90] Ervin RB, Kit BK, Carroll MD, Ogden CL. Consumption of added sugar among U.S. children and adolescents, 2005–2008. NCHS Data Brief. 2012;(87):1–8. . 22617043

